# Cross-border referral for early breast cancer: an analysis of radiation fractionation patterns

**DOI:** 10.3390/curroncol13040013

**Published:** 2006-08

**Authors:** I.S. Dayes, T.J. Whelan, J.A. Julian, M.R. Kuettel, D. Regmi, G.S. Okawara, M. Patel, H.I. Reiter, S. Dubois

**Affiliations:** * Department of Radiation Oncology, Juravinski Cancer Centre; and Department of Medicine, McMaster University, Hamilton, Ontario; † Supportive Cancer Care Research Unit, McMaster University, Hamilton, Ontario.; ‡ Department of Clinical Epidemiology and Biostatistics, McMaster University, Hamilton, Ontario.; § Department of Radiation Medicine, Roswell Park Cancer Institute; and Department of Radiation Oncology, School of Medicine and Biomedical Sciences, University at Buffalo, Buffalo, New York.; || Research Department, St. Joseph’s Care Group; and Centre for Education and Research on Aging and Health, Lakehead University, Thunder Bay, Ontario

**Keywords:** Breast cancer, cross-border referral, fractionation, patterns of practice, radiotherapy

## Abstract

Because of increasing waiting times for adjuvant radiation in the province of Ontario, patients from one Canadian centre were referred to two centres in the United States. This situation provided an opportunity to compare radiation practices.

We performed a retrospective review of radiation prescribed to patients following breast-conserving surgery for invasive breast cancer. Patients with positive margins, 4 or more positive lymph nodes, recurrent disease, or large tumours (>5 cm) were excluded. For comparison, we reviewed a random sample of similar patients treated at the Canadian centre during the same period. A total of 120 referred and 217 non-referred patients were eligible for comparison. The analysis included 98 pairs of patients (*N* = 196), fully matched on age, nodal status, T stage, grade, and estrogen receptor (er) status.

Mean patient age was 60.7 years. The median total dose and number of fractions differed between centres [6040 cGy in 32 fractions (United States) vs. 4250 cGy in 16 fractions (Canadian), both *p* < 0.001). Boost was used more often in the United States (97% vs. 9%, *p* < 0.001). Variation in prescribing patterns was seen. In the United States, seven different schedules for whole-breast irradiation were used; at the Canadian centre, two schedules were prescribed. Predicted radiobiologic effects of these schedules were calculated to be similar.

Differences in fractionation patterns were observed between and within U.S. and Canadian centres. Such variability is likely to affect patient convenience and resource utilization. Although patient selection, referring surgeon, and change in policies may account for some of the observed differences, further research is necessary to better understand the causes.

## 1. INTRODUCTION

Considerable variation in radiation therapy fractionation patterns following breast-conserving surgery has been documented in several health care regions 1–4. In the past, individual radiotherapy centres developed their own fractionation regimens empirically, and those patterns have persisted. In addition, several pivotal randomized trials that established the role of breast irradiation following breast-conserving surgery used various fractionation schedules 5–7. Few clinical studies that compare fractionation schedules have been performed, but evidence supports the probable biologic equivalency of the commonly used schedules with respect to local control and toxicity 4,8,9. Variation in treatment schedules may have a significant effect on treatment resources 10 and patient convenience.

Potential sources of variation include standard schedules that vary between clinics, the training and preferences of the treating physician, patient preference and traveling distance, and resource constraints. Typically, reports of variation have examined patients treated in different centres. However, comparison across centres may be problematic, because patients are referred from different pools of referring surgeons. Differences in local surgical trends may account for some of the observed variation in radiation schedules.

In the 1990s, significant delays for radiation therapy treatment were becoming increasingly recognized in Canada 11,12. Because of increasing waiting times for breast irradiation in Ontario in early 1999, a process of re-referral to other centres was developed to expedite care. At the time of consultation, eligible patients were made aware of extended wait times and offered referral to another centre, primarily across the border in the United States. Consenting patients were referred for a second radiation oncology consultation at a U.S. centre, where treatment was given. This program provided a unique opportunity to compare patterns of treatment at various centres in Canada and the United States, among patients drawn from the same surgical population.

## 2. MATERIALS AND METHODS

All patients were initially seen for radiation consultation at a single Ontario institution. During the study period, the annual number of breast cancer patients being seen for radiation therapy was approximately 700. Patients were referred by surgeons from all communities within the central-west area of Ontario, where the one institution under study is the only site for radiation delivery. Limited machine availability led to extended delays for radiation therapy, and therefore all eligible patients were offered re-referral to participating centres in the United States. The U.S. centres had previously been selected after site visits from Ontario radiation oncologists and assessments of physics quality assurance and clinical activity. Patients were eligible if they were well enough to travel independently, were not primary caregivers for others, and had no distant metastases. Provincial authorities covered the referred patients’ personal costs for transport and accommodation.

Clinic computer records were used to identify breast cancer patients who had undergone re-referral. In addition, a list of breast cancer patients treated during the same period but not referred to U.S. centres was obtained. From that list, a random group with oversampling was chosen to provide a comparison group. A standardized data collection form was used to record patient data; surgery and pathology details; use of systemic treatment; and details of radiotherapy, including dose, fractionation, energy, use of boost, and planning technique. Patients were identified solely by initials and chart number.

Patients were eligible for the comparison group if they had undergone breast-conserving surgery with complete resection for early-stage invasive breast cancer (T1–T2, N0–N1, M0). The referral program was active from April 1999 until March 2001. Midway through the referral process (July 2000), the U.S. centres began to treat referred patients according to the fractionation policy at the Canadian centre, at the request of Ontario’s provincial cancer organization. It was felt that inclusion of patients who received treatment after that date would limit the validity of the present study’s observations, and so those patients were excluded. From among the eligible patients, two groups were created: patients treated in Canada were paired with patients treated in the United States, fully matched on five criteria. Those criteria were age category (<40 years, 40–70 years, >70 years), axillary node status (negative, 1–3 nodes positive), T stage (T1, T2), tumour grade (1, 2, 3), and estrogen receptor (er) status (positive, negative).

Because of the nonparametric nature of the collected data, the Wilcoxon rank sum test was used to compare dose and fractionation for whole-breast and boost irradiation. Matching had maximized the similarity between the two cohorts with respect to prognostic factors. Because of the constant matching ratio (1:1) and the use of historical cohorts (rather than a case–control approach), little or no expected loss of efficiency would occur by breaking the match 13. The analysis was therefore performed in an unmatched fashion. Chi-square analysis was used to test differences in proportions. Conover’s nonparametric test was used to compare variance 14. The significance level was set at *p* < 0.05, two-sided.

## 3. RESULTS

Details of all eligible referred patients were available for analysis. Matching on all five pre-selected criteria (age, T stage, node status, grade, er status) to a randomly selected group of patients seen during the same period but not referred for treatment elsewhere resulted in 196 patients available for comparison. The patient groups were very similar with respect to known prognostic factors (Table I).

Comparison of the matched groups revealed differences in prescribed radiotherapy with respect to dose and number of fractions for whole-breast and boost treatments (Tables II–IV). In the referral group, seven different whole-breast fractionation schedules were used; in the comparison group, two different schedules were used (difference in variance: *p* < 0.001). Whole-breast doses to patients treated in the United States ranged from 4000 cGy to 5040 cGy, and no single fractionation schedule was given to more than 40% of patients. In the group treated in Canada, 92% of patients were treated with 4250 cGy in 16 fractions; all others were treated with 5000 cGy in 25 fractions. Median prescribed doses were significantly higher in the United States [5000 cGy (range: 4000–5040 cGy) vs. 4250 cGy (range: 4250–5000 cGy), *p* < 0.001]. Median number of fractions given was also higher in the group treated in the United States [25 fractions (range: 20–28 fractions) vs. 16 fractions (range: 16–25 fractions), *p* < 0.001].

Boost irradiation was given in the U.S. centres to 97% of patients, using five different schedules. In the Canadian centre, only 9% of patients received a boost, with two schedules being used (*p* < 0.001). Again, fractionation patterns were different between the groups. In patients receiving boost, the median boost dose delivered was 1400 cGy in the United States (range: 900–1600 cGy) and 1000 cGy in Canada (range: 900–1000 cGy). Boost was delivered over a median of 5 fractions in both centres, but differences were observed in the ranges [5–15 fractions (United States) vs. 4–5 fractions (Canada)].

**FIGURE 1:**
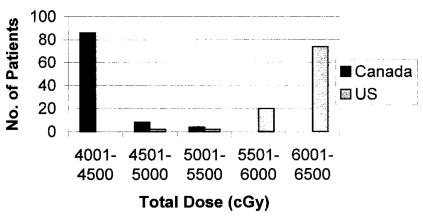
Differences in total dose delivered by each centre.

Median total dose (whole-breast plus boost) was higher in the group treated in the United States [6040 cGy (range: 5000–6500 cGy) vs. 4250 cGy (range: 4250–5250 cGy), *p* < 0.001], with few patients receiving less than 6000 cGy (Figure 1). Median number of prescribed fractions was also higher in the group treated in the United States [32 fractions (range: 25–39 fractions) vs. 16 fractions (16–25 fractions), *p* < 0.001].

## 4. DISCUSSION AND CONCLUSIONS

Patterns of care for breast cancer have previously been noted to vary between practice areas 2,4,10. Our report summarizes treatment characteristics in two similar groups of patients from the same referral base, treated in different centres in different countries. The referred cohort treated in the United States was compared to a randomly selected control group of patients with matched prognostic factors who were treated in Canada. Findings for the referred group’s treatment are in keeping with previously reported U.S. data with respect to whole-breast dose, boost dose, and proportion of patients receiving boost 15. In the patients treated in Canada, the median whole-breast dose was similar to that reported in a previous study of breast irradiation delivered in Ontario 3.

Several factors may account for the observed differences between the groups. Previously identified factors include local policy, training, patient convenience, and differences in clinical trials supporting the use of adjuvant breast irradiation 1,10. National guidelines and resources both likely played a large part in the radiation schedules seen in the study. The guideline published in the *Canadian Medical Association Journal* 16 supports the use of either longer or shorter fractionation schedules, but recommends shorter schedules when resources are limited. Although this Canadian guideline does not suggest the routine use of boost, the National Comprehensive Cancer Network (nccn) guideline recommends boost to the tumour bed. Interestingly, the nccn guideline does not discuss fractionation schedules or total dose 17. Within each centre, adherence to the relevant national radiotherapy guidelines appears high.

The strength of this study is its homogeneous pool of patients treated by the same referring surgeons, available for data collection and matching, with few missing data, for a unique cross-border comparison. This common pool of patients served to reduce some of the limitations identified in previous studies of breast cancer practice patterns 18. To standardize the non-randomized comparison groups, inclusion for analysis was based upon restrictive criteria, with matching performed on the full set of five pre-selected prognostic factors. This strict pairing left some records unmatched, but the final comparison was based on 196 patients in two very similar groups. Analysis of all eligible patients (*n* = 337), including those not matched, did not alter the study’s findings (data not shown).

In this study we observed marked differences with respect to fractionation schedules to the whole breast and the boost to the primary surgical site. The most commonly used whole-breast schedule was 42.5 Gy in 16 fractions in the Canadian centre and 50.4 Gy in 28 fractions in the U.S. centres. Boost irradiation was infrequently used in the Canadian centre and commonly used in the U.S. centres, generally to a higher dose. We also observed a higher number of different fractionation schedules in use in the U.S. centres as compared with the Canadian centre, both to the whole breast and to the surgical site.

A substantial variation between the centres in the fractionation schedules used is not surprising. Several different schedules were used in the randomized trials that originally demonstrated the effectiveness of breast irradiation, and as a result, all of the schedules are considered acceptable for the treatment of women following breast-conserving surgery.

Interestingly, despite the differences in total dose and number of fractions between these schedules, all are predicted to have a similar biologic effect. The effect of radiation on tumour control and normal tissue is directly related to total dose and fraction size. Tumour control also appears to be adversely related to the overall length of treatment, so that shorter treatments are predicted to be more effective. Thus, radiobiologic models suggest that a dose of 50.4 Gy in 28 fractions given over 5.5 weeks is likely to be similar in effect to a dose of 42.5 Gy in 16 fractions given over 3 weeks. In this instance, although the overall dose is lower, the dose per fraction is increased, and the overall treatment time is shortened by almost 50%. Recent randomized data support these radiobiologic models, suggesting that a slightly lower dose given in 3 weeks is just as effective as a higher dose given in 5 weeks, both with respect to tumour control and radiation morbidity in normal tissue 8. Hopefully, optimal treatments can be determined as more data become available on the radiobiology of breast tissue and the effect of fractionation 19.

The advantages of the shorter radiation schedule are primarily patient convenience and cost of treatment where health care is provided by a public system, as in Canada. As waiting times grow because of resource limitations, shorter schedules become more attractive. In the United States, where health care is provided both by public and by private systems, resources for radiation are less limited and more easily accessible; the demand for shorter radiation schedules is less. In Canada, radiation therapy is provided in regionalized centres, making the therapy less accessible to patients living in rural areas.

In this study we also observed differences in the use of boost irradiation to the primary site following whole-breast irradiation. Again, this finding is not surprising. The use of boost irradiation in patients with clear margins of excision following breast-conserving surgery has been controversial. It is often difficult to localize the surgical cavity for additional radiation, and during the study period, it was unclear whether such treatment is effective. Boost irradiation has been routinely used in some institutions, but it was not consistently used in the randomized studies that established the role of adjuvant breast irradiation 6,20. Later, a large European randomized trial demonstrated that boost irradiation is effective 21. The benefit appears to be substantial for younger women, but at the expense of increased morbidity to the skin and soft tissue, resulting in a decreased cosmetic outcome. The effect of boost irradiation for older women appears to be limited. This European trial was not published before the treatment period evaluated in the present study. The limited evidence of effectiveness of boost, in conjunction with limited resources, probably made boost irradiation less attractive in Canada. With the publication of the European trial, that situation has probably changed.

The limitations of our study include its retrospective nature and its generalizability, which may be limited because it is based on results from only three centres (one in Ontario and two in New York State), rather than on a comparison of the entire province and the entire state. In addition, comparing a single institution to two others may have inflated estimates in differences of variability. There is also concern that the mere creation of the referral program may have influenced fractionation practices. For example, referred patients may have created a resource strain in the U.S. centres and reduced the strain in the Canadian one, which may have influenced prescribing patterns and thus potentially limited the differences observed.

Within a similar patient population, this analysis revealed several differences in the radiation prescribing patterns in U.S. and Canadian cancer centres during a cross-border referral program designed to alleviate waiting times for treatment for breast cancer. The differences were likely generated by multiple factors—in particular, physician training, institutional preference, and adherence to different national guidelines—and reflect the variation in the schedules used in the randomized trials that demonstrated the effectiveness of the treatment. Pressures from different health care systems were also likely to influence the choice of fractionation schedule. More recent randomized trials demonstrated the equivalence of various fractionation schedules and the importance of using boost irradiation in selected patients. Such studies are likely to have an influence in the choice of fractionation schedules used both north and south of the border. Because cross-border referral programmes may continue to be a strategy to expedite patient care in Canada 22, further study and a better understanding of these factors may be of value when creating local policies, allocating health resources, and conducting future clinical studies.

Issues of patient convenience and resource considerations are becoming increasingly important for all countries as the incidence of early breast cancer continues to increase because of an aging population and detection by screening mammography. As a result, there is increasing interest in shortening treatment schedules even further with the use of accelerated partial-breast irradiation. The rationale for this approach is based on the observation that more local recurrences occur at the primary surgical site. By limiting treatment to a smaller area, radiation can be given in shorter treatment periods ranging from 1 day to 1 week. Such treatments are also likely to be more cost-effective. As a result, the variation in fractionation schedules used for whole-breast irradiation are expected probably to decrease both within and between different countries over time. Such uniformity is desirable for women if it results in more convenient, more effective, and less costly treatment.

## Figures and Tables

**TABLE I:** Clinical characteristics of the matched groups (*n*=98, each group)

	*Group*	
*Characteristic*	*Control*	*Referral*
Mean age (years)	60.4	61.1
Mean tumour size (mm)	15.0	13.9
Grade iii^a^ (%)	20.4	20.4
Node-negative a (%)	92.9	92.9
er positive a (%)	86.7	86.7
Left-sided b,c (%)	56.1	45.9
Axillary dissection b (%)	70.4	72.4
Nodes removed ≥ 10 b (%)	49.3	47.9

a Matching was done by exact category; therefore percentages are expected to be identical. See text.

b Not used as a matching criterion.

c *p* = 0.08; all other comparisons *p* > 0.1.

er = estrogen receptor.

**TABLE II:** Number of patients receiving specified whole-breast fractionation schedules

	*Group*	
*Schedule (cGy/fractions)*	*Control*	*Referral*
4000/20	0	1
4250/16	90	0
4500/25	0	2
4600/23	0	17
4680/26	0	1
4800/24	0	7
5000/25	8	34
5040/28	0	36

**TABLE III:** Boost doses delivered

	*Group (*n *a)*	
*Dose (cGy)*	*Control*	*Referral*
0	85	1
900	1	1
1000	8	34
1400	0	36
1500	0	21
1600	0	1

a Boost data not available on all 196 patients.

**TABLE IV:** Treatment differences found between groups

	*Group*	
*Prescription factor*	*Control*	*Referral*
Median dose to whole breast [cGy (range)]	4250 (4250–5000)	5000 (4000–5040)
Median boost dose [cGy (range)] a	1000 (900–1000)	1400 (900–1600)
Median total dose [cGy (range)]	4250 (4250–5250)	6040 (5000–6500)
Median fractions to whole breast [*n* (range)]	16 (16–25)	25 (20–28)
Median boost fractions [*n* (range)] a	5 (4–5)	5 (5–15)
Median total fractions [*n* (range)]	16 (16–25)	32 (25–39)
Patients receiving boost (*n*/*N*) b	9/98	95/98
Patients receiving loco-regional radiation (*n*/*N*) b,c	0/98	7/98

a Median boost dose and fractions only of those patients who received a boost, not the entire group. Therefore, totals do not reflect the sums of whole-breast and boost numbers.

b Difference tested using Fisher exact test; all others tested using Wilcoxon rank sum test.

c *p* = 0.01; all other comparisons, *p* < 0.001.
